# CD56+ monocytes have a dysregulated cytokine response to lipopolysaccharide and accumulate in rheumatoid arthritis and immunosenescence

**DOI:** 10.1186/ar4321

**Published:** 2013-10-01

**Authors:** Marco Krasselt, Christoph Baerwald, Ulf Wagner, Manuela Rossol

**Affiliations:** 1 Division of Rheumatology, Department of Internal Medicine, University of Leipzig, Liebigstrasse 20, D-04103 Leipzig, Germany

## Abstract

**Introduction:**

Peripheral blood monocytes are no longer regarded as a homogeneous cell population, but can be differentiated both phenotypically and functionally into various subpopulations. In rheumatoid arthritis, the subpopulation of CD14^bright^/CD16+ monocyte is expanded and prone towards generation of Th17 cells. CD56+ monocytes represent a different subpopulation, which is also expanded in conditions associated with autoimmunity like inflammatory bowel diseases. The aim of the study was the quantification and functional characterization of the CD56+ monocyte subset in rheumatoid arthritis (RA).

**Methods:**

Frequencies of peripheral blood monocyte subpopulations were analyzed by flow cytometry in 86 healthy controls and 75 RA patients. In 16 patients, anti-tumor necrosis factor (TNF) therapy was initiated, and the CD56+ monocyte frequency was monitored longitudinally. Lipopolysaccharide (LPS)-induced cytokine production of CD56+ and CD56– monocytes was determined by intracellular staining or cytokine secretion assays.

**Results:**

In healthy individuals, 8.6% ± 0.6 of the monocytes co-expressed CD56, with the majority of CD56+ monocytes being CD14^bright^ (7.9% ± 0.5), while only a minor population was CD14^dim^ (0.7% ± 0.1). We found a strong positive correlation between an individual’s age and the frequency of CD56+ monocytes. Upon stimulation with LPS, CD56+ monocytes became more frequently positive for TNF, IL-10 and IL-23 than CD56– monocytes. In addition, CD56+ monocytes spontaneously produced more reactive oxygen intermediates than CD56- monocytes. In RA patients, the frequency of CD56+ monocytes was significantly higher than in healthy controls (12.2% ± 0.9 vs. 7.9% ± 0.5, p = 0.0002), and this difference most pronounced in RA patients below 40 years of age (11.1% ± 1.6 vs. 4.1% ± 0.4, *P* < 0.0001). Treatment of the patients with an anti-TNF blocking agent significantly reduced CD56+ monocyte frequencies (baseline 12.4% vs. 24 weeks treatment 8.0%, *P* = 0.0429), and the magnitude of this decrease was found to correlate with the change in disease activity under the therapy.

**Conclusion:**

The CD14^bright^/CD56+ monocyte subset is expanded in aging individuals as well as in patients with RA. The pro-inflammatory production of cytokines and reactive oxygen species as well as the elimination of those cells in patients with a good response towards TNF inhibiting agents indicates a possible contribution of those monocytes in the inflammatory response in RA.

## Introduction

Peripheral blood monocytes are not a homogeneous cell population, but represent different subpopulations with distinct functions and cell surface markers. Three major subpopulations can be distinguished by the expression of the cell surface markers CD14 and CD16, classical CD14^bright^CD16– monocytes, nonclassical CD14^dim^CD16+ monocytes and intermediate CD14^bright^CD16+ monocytes [1]. More recently, a separate and less well-characterized monocyte subpopulation has been described which is characterized by the expression of the neural cell adhesion molecule CD56 [2]. CD56+ monocytes are found in low frequencies in the peripheral blood of healthy individuals [2,3], patients with Down syndrome [4] and patients with chronic myelomonocytic leukemia [5]. This monocyte subpopulation is expanded in Crohn’s disease [3], produces typical monocyte cytokines [2] and is a more efficient antigen-presenting cell population with regard to the induction of a T-cell alloresponse [2].

It is already known that the monocyte compartment is disturbed in patients with rheumatoid arthritis (RA). We and others have observed an increase in the frequency of CD16-expressing monocytes [6-8]. To date, no studies evaluating the presence of CD56+ monocytes have been performed in RA patients.

Herein we report an increased frequency of CD56+ monocytes in patients with RA compared to healthy controls. The occurrence of CD56+ monocytes in the peripheral blood is strongly age-dependent in healthy controls, but this association is lost in RA patients. CD56+ monocytes produce more tumor necrosis factor (TNF), interleukin 10 (IL-10) and IL-23 than CD56– monocytes, and anti-TNF therapy normalizes the frequency of CD56+ monocytes in RA patients.

## Methods

### Human participants

Seventy-five patients with RA were included in the study. The diagnosis of RA was based on the American College of Rheumatology/European League Against Rheumatism 2010 classification criteria for RA [9]. Sixteen patients required therapy with a TNF-blocking agent because of their uncontrolled disease, and therefore etanercept treatment was initiated while concomitant disease-modifying antirheumatic drug therapy was continued. The dynamics of the CD56+ monocyte population were monitored before the initiation of therapy and during the following 24 weeks. The characteristics of the study populations are shown in Table 1.

**Table 1:** Characteristics of the rheumatoid arthritis patient cohorts

**Characteristics**	**All RA patients**	**Longitudinal etanercept study**
	**(*N*** **= 75)**	**(*N*** **= 16)**
Median age, years (range)	57.0 (23 to 83)	56.0 (41 to 66)
Female/male (*n*)	55/20	10/6
Median disease duration, years (range)	7.0 (1 to 57)	2 (1 to 13)
RF-positive (%)	77.0	68.8
Anti-CCP-positive (%)	75.8	93.8
Median CRP, mg/dl (range)	3.53 (0 to 113)	4.40 (0.5 to 21.1)
DMARDs (*n*)		
Methotrexate	42	13
Azathioprine	1	0
Leflunomide	4	1
Anti-TNF	7	0
Anti-TNF + MTX	2	0
Abatacept	1	0
Hydroxychloroquine + MTX	1	1
Cyclosporin A	1	0
Cyclosporin A + MTX	1	0
Tocilizumab	1	0
Rituximab	1	0
Without	13	1

^a^CCP, cyclic citrullinated peptide; CRP, C-reactive protein; DMARDs, disease-modifying antirheumatic drugs; MTX, methotrexate; RA, rheumatoid arthritis; RF, rheumatoid factor; TNF, tumor necrosis factor.

Eighty-six control subjects were recruited among healthy blood donors (median age 53.5 years, range 22 to 72 years; 33 males and 53 females). The ethics committee of the University of Leipzig approved all experiments with human materials, and informed consent was obtained from all participants.

### Cell isolation and culture

Human peripheral blood mononuclear cells (PBMCs) were isolated as described previously [10]. Cells were incubated in RPMI 1640 medium supplemented with 10% heat-inactivated fetal calf serum, 2 mM L-glutamine, 100 U/ml penicillin and 100 μg/ml streptomycin at a concentration of 1 × 10^6^/ml.

### Flow cytometry

To identify monocyte subsets in the peripheral blood, PBMCs were stained with anti-CD56-allophycocyanin (AF12-7H3), anti-CD14-fluorescein isothiocyanate (TÜK4) and anti-CD16-phycoerythrin (VEP13). Antibodies were obtained from Miltenyi Biotec (Bergisch Gladbach, Germany). Cells were analyzed using a FACSCalibur flow cytometer and CellQuest software (BD Biosciences, San Jose, CA, USA).

### Measurement of cytokine production

PBMCs were stimulated for four hours (to measure TNF production) or sixteen hours (to measure IL-10, IL-23 and IL-1β production) with 100 ng/ml ultrapure lipopolysaccharide (LPS) (InvivoGen, Toulouse, France) or were left untreated. Subsequently, cells were harvested and TNF-producing or IL-10-producing monocytes were identified using cytokine secretion assays according to the manufacturer’s protocol (Miltenyi Biotec). To determine IL-23 and IL-1β production, BD GolgiStop was added and intracellular cytokine staining was performed using BD Cytofix/Cytoperm Fixation/Permeabilization Solution Kit (Becton Dickinson GmbH, Heidelberg, Germany) and anti-IL-23 (eBioscience, San Diego, CA, USA) or anti-IL-1β (R&D Systems, Minneapolis, MN, USA) antibodies. Cells were analyzed using a FACSCalibur flow cytometer and CellQuest software.

### Reactive oxygen intermediate measurement

PBMCs were stimulated for up to two hours with 100 ng/ml ultrapure LPS or 20 ng/ml phorbol 12-myristate 13-acetate (PMA) or were left untreated. To detect reactive oxygen intermediates (ROIs), dihydrorhodamine 123 was added to the living cells and fluorescence was monitored using a FACSCalibur flow cytometer and CellQuest software.

### Statistical analysis

To determine statistical significance, Student’s *t*-test or the Mann–Whitney rank-sum test was performed. Prior to all comparisons, the Kolmogorov-Smirnov normality test was done.

## Results

### Frequency of CD56+ monocytes increases throughout life in healthy controls

**Figure 1:**
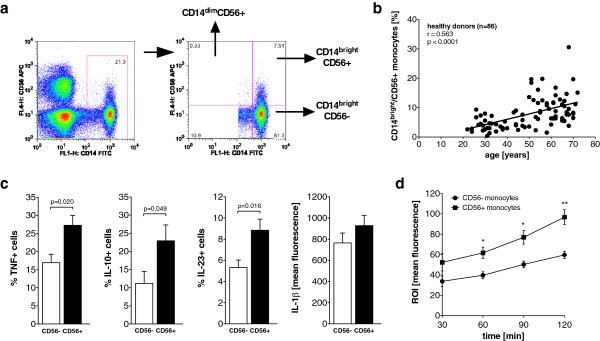
**CD14**^**bright**^**/CD56+ monocyte subset is expanded in older healthy controls and produces more cytokines in response to lipopolysaccharide. (a)** Representative dot plot of CD14 and CD56 expression on monocytes. Three relevant subpopulations are separated by the quadrants and marked by the arrows in the right panel. APC, allophycocyanin; FITC, fluorescein isothiocyanate; FL1-H, fluorescence intensity on channel that detects emissions from fluorescein isothiocyanate. **(b)** Scatterplot showing the correlation between age and the peripheral blood frequencies of CD14^bright^/CD56+ monocytes in healthy controls. **(c)** Bar graphs depict the frequency of tumor necrosis factor-positive (TNF+) (*n* = 5), interleukin 10-positive (IL-10+) (*n* = 8) and IL-23+ (*n* = 7) cells and the mean intracellular IL-1β content (*n* = 7) in CD56+ and CD56– monocytes of healthy controls in response to lipopolysaccharide. **(d)** Spontaneous reactive oxygen intermediate (ROI) production of CD56– and CD56+ monocytes (*n* = 4).

CD56+ monocytes were identified in PBMCs by the coexpression of CD56 and the monocytic surface marker CD14 (Figure 1a). Eighty-six healthy controls were analyzed, and 8.6% ± 0.6 of the monocytes coexpressed CD56. Most of the CD56+ monocytes are CD14^bright^ (7.9% ± 0.6), and only a minor population is CD14^dim^ (0.7% ± 0.1). Based on the scatter properties in flow cytometric analyses, the CD14^bright^/CD56+ monocyte subset had the appearance of classical monocytes, whereas the CD14^dim^/CD56+ monocyte subset resembled large lymphocytes and natural killer (NK) cells (data not shown).

All subsequent analyses were performed on CD14^bright^/CD56+ monocytes in comparison to CD14^bright^/CD56– monocytes, unless stated otherwise.

It is already known that CD14^bright^ monocytes can be subdivided into two subpopulations by the expression of the surface marker CD16 [1], representing classical monocytes (CD14^bright^/CD16–) and intermediate monocytes (CD14^bright^/CD16+). CD16 and CD56 expression was coanalyzed in ten healthy controls. CD56+ monocytes were found to be mostly CD16– and therefore to belong to the classical CD14^bright^/CD16– monocytes. Only 7.5% ± 1.6 of the CD56+ monocytes coexpressed CD16 and belonged to the intermediate CD14^bright^/CD16+ monocytes.

To analyze the influence of age on the CD56+ monocyte subpopulation, the control cohort was recruited to reflect a wide age range (median age 53.5 years, range 22 to 72 years). The analysis revealed a close positive correlation between age and CD14^bright^/CD56+ monocyte frequency (Figure 1b), as well as between age and CD14^dim^/CD56+ monocyte frequency (*r* = 0.332, *P* = 0.0018). No difference in CD56+ monocyte frequencies between women and men were discernible (data not shown).

### CD14^bright^/CD56+ monocytes show an increased cytokine production and spontaneous reactive oxygen intermediate production

Little is known about the cytokine production of CD56+ monocytes. Sconocchia *et al*. performed a cytokine array analysis [2], showing that CD56+ monocytes produce typical monocytic cytokines, but no *in vitro* studies of cytokine production of CD56+ monocytes in comparison to CD56– monocytes have been performed.

A cytokine secretion analysis in monocytes of young healthy controls revealed that increased frequencies of cells producing the proinflammatory cytokines TNF and IL-23 and the anti-inflammatory cytokine IL-10 in response to LPS were found in CD14^bright^/CD56+ monocytes compared to CD14^bright^/CD56– monocytes (Figure 1c). In contrast, intracellular staining for IL-1β showed a similar cytokine production of both CD14^bright^ monocyte subsets in response to LPS (Figure 1c). No difference in the spontaneous production of cytokines was observed (data not shown).

The production of ROIs in response to bacterial products is a characteristic feature of monocytes. No difference in LPS-induced ROI production was observed between CD14^bright^/CD56+ monocytes and CD14^bright^/CD56– monocytes (data not shown). The same result was obtained when the production of ROIs was initiated by direct stimulation of protein kinase C with PMA (data not shown). However, the spontaneous production of ROIs was higher in CD14^bright^/CD56+ monocytes than in CD14^bright^/CD56– monocytes (Figure 1d).

### Frequency of CD14^bright^/CD56+ monocytes is increased in young rheumatoid arthritis patients

To analyze the CD56+ monocyte subpopulation in patients with RA, 75 patients with a median age of 57.0 years (range 23 to 83 years) were recruited. For a detailed clinical description of the patient cohort, see Table 1. Of the RA monocytes, 13.1% ± 0.9 coexpressed CD56, 12.2% ± 0.9 were CD14^bright^ and 0.9% ± 0.1 were CD14^dim^.

**Figure 2:**
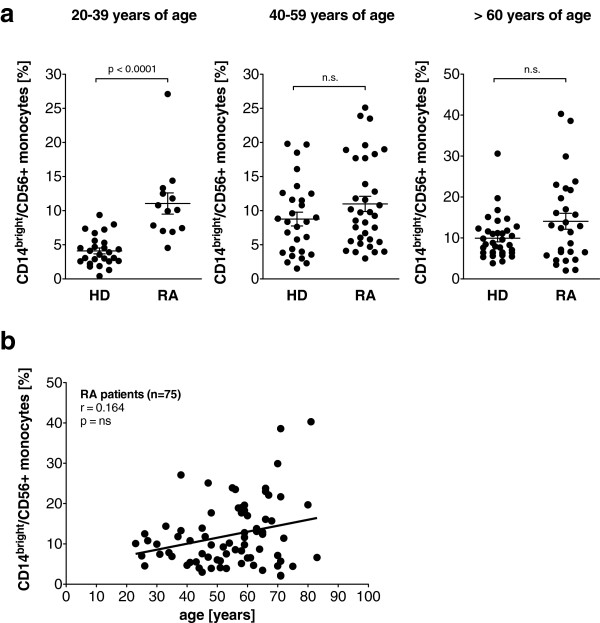
**CD14**^**bright**^**/CD56+ monocyte subset is expanded in young rheumatoid arthritis patients. (a)** Peripheral blood frequencies of CD14^bright^/CD56+ monocytes in healthy controls (HD) and rheumatoid arthritis patients (RA). **(b)** Correlation between age and the peripheral blood frequencies of CD14^bright^/CD56+ monocytes in RA patients.

In the global analysis, RA patients had a higher frequency of CD14^bright^/CD56+ monocytes than healthy controls (12.2% ± 0.9 vs. 7.9% ± 0.5; *P* = 0.0002). The CD14^dim^/CD56+ monocyte subset was also found to be expanded in RA patients (0.9% ± 0.1 vs. 0.7% ± 0.1; *P* = 0.029), although the difference was less pronounced. In view of the strong dependence of the CD56+ monocyte frequency on age in the control cohort, the RA patients and controls were separated into three different age subsets (20 to 39 years, 40 to 59 years and 60 years and older). As shown in Figure 2a, the increase of CD14^bright^/CD56+ monocyte frequencies in the RA patients was limited to the age subset from 20–39 years, while frequencies in older RA patients did not differ significantly from age-matched controls. The CD14^dim^/CD56+ monocyte subset was also found to be expanded in young RA patients (0.6% ± 0.1 vs. 0.8% ± 0.1, p = 0.046) but not in older patients (data not shown). In the global analysis of the total RA cohort, no correlation of age with the CD14^bright^/CD56+ monocyte subset was found (Figure 2b).

Analysis of the disease duration prior to study enrollment revealed no correlation with the CD14^bright^/CD56+ monocyte frequency (data not shown). The CD14^bright^/CD56+ monocyte subset is already expanded early in the disease because RA patients with a disease duration of one year have 12.0% ± 1.8 CD14^bright^/CD56+ monocytes compared to 12.3% ± 1.1 CD14^bright^/CD56+ monocytes in patients with a longer disease duration (*P* = n.s.). The therapeutic regimen at the time of the analysis, the presence of rheumatic factor or anti-CCP antibodies, the absolute monocyte count, C-reactive protein levels and gender had no influence on CD14^bright^/CD56+ monocyte frequency.

### Therapeutic tumor necrosis factor blockade with etanercept decreases frequency of CD14^bright^/CD56+ monocytes

To analyze the influence of treatment and of the therapeutic response on the frequency of the expanded CD14^bright^/CD56+ monocyte subset, the cell population was quantified in a longitudinally followed cohort of 16 RA patients before and during 24 weeks of treatment with etanercept. Before the start of treatment, RA patients had a mean CD14^bright^/CD56+ monocyte frequency of 12.4% ± 1.7, and no correlation of disease activity with the frequency of CD14^bright^/CD56+ monocytes was detectable (data not shown).

**Figure 3:**
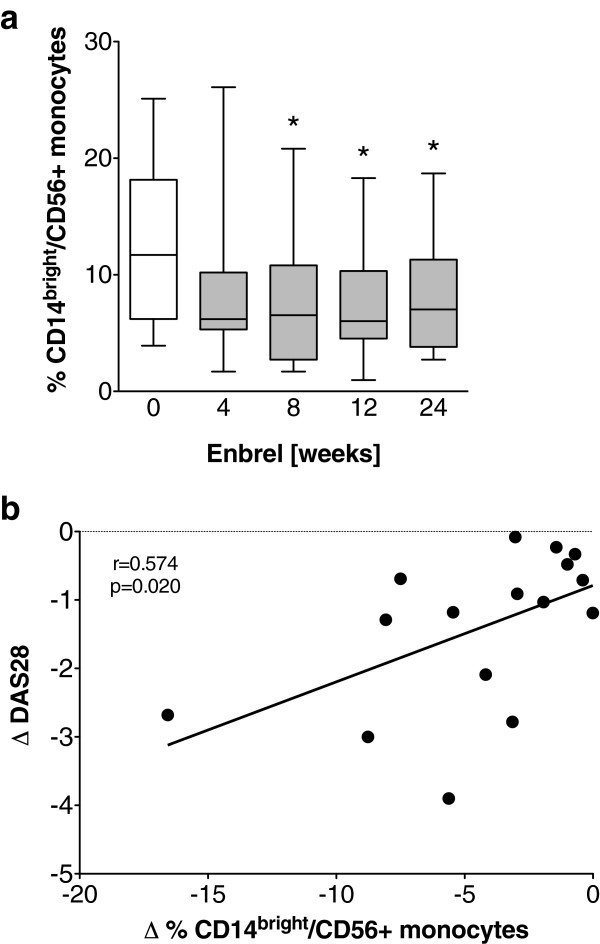
**CD14**^**bright**^**/CD56+ monocyte subset decreases during therapeutic tumor necrosis factor blockade. (a)** Peripheral blood frequencies of CD14^bright^/CD56+ monocytes in 16 RA patients before and after treatment with etanercept. **(b)** Change in Disease Activity Score in 28 joints (∆DAS28) and CD14^bright^/CD56+ monocyte frequency at week 12 of treatment with etanercept (Enbrel) correlated to baseline (*n* = 16).

In the prospective analysis, the frequency of CD14^bright^/CD56+ monocytes in this patient cohort was found to decrease after eight weeks of treatment and remained decreased for up to twenty-four weeks, which was the endpoint of the study (Figure 3a). Interestingly, RA patients with a good response to the treatment also showed a more pronounced decrease in the CD14^bright^/CD56+ monocyte frequency after 12 weeks (Figure 3b) and 24 weeks (data not shown) of treatment.

## Discussion

To date, the CD56+ monocyte subpopulation has not been very well-characterized. Although CD56+ monocytes were found in 2001 in patients with Down syndrome with or without an additional hematological disorder [4], Sconocchia *et al*. first described CD56+ monocytes in the peripheral blood of healthy controls in detail in 2005 [2]. However, the same study group had previously described the differentiation of CD56+ monocytes from CD34+ stem cells in 2004 [11]. Herein we show that the frequency of CD56+ monocytes dramatically increases with age in healthy controls; that CD56+ monocytes produce more TNF, IL-10 and IL-23; that CD56+ monocytes are expanded in young RA patients; and that the CD56+ monocyte subset responds to anti-TNF treatment in RA patients.

We were able to identify two subsets of CD14+ monocytes coexpressing CD56 in the peripheral blood of healthy controls and RA patients. The CD14^bright^/CD56+ monocyte subset represents the main CD56+ monocyte population, and these cells had the same flow cytometry scatter appearance as classical monocytes. In contrast, the minor CD14^dim^/CD56+ monocyte subset mostly had the appearance of lymphocytes and NK cells, which probably corresponds to the CD56+ cell population described by Gruenbacher *et al*. [12]. This group described CD56+ cells in the peripheral blood, which are CD14^dim^, HLA-DR+ and CD86+; have the appearance of intermediate-sized lymphocytes; and differentiate *in vitro* into dendritic cells. Sconocchia *et al*. also described a CD56+/CD33+ myeloid cell population which is able to differentiate *in vitro* into cells with a dendritic cell morphology [13]. Although the two CD14^bright^/CD56+ and CD14^dim^/CD56+ populations have different size and granularity proportions, it remains to be established whether these populations are truly two different monocyte subsets or if they belong to one CD56+ monocyte subset. Some of our observations point to the latter possibility because both CD14^bright^/CD56+ and CD14^dim^/CD56+ populations increase comparably with age in healthy controls and are equally expanded in RA patients.

We observed only a minimal overlap of the CD14^bright^/CD56+ monocyte subset with the well-characterized CD14^bright^/CD16+ intermediate monocyte subset (7.5% of the CD56+ monocytes coexpressed CD16). Contamination with NK cells can be excluded because the cells expressed high levels of the monocytic lineage marker CD14. CD14^bright^/CD16+ intermediate monocytes are the main producer of IL-1β, TNF and IL-23 in response to LPS [14]; are expanded in RA patients [6]; and promote the expansion of Th17 cells [6]. Most of the CD14^bright^/CD56+ monocytes belong to the classical CD14^bright^/CD16– monocyte subset, which is the main producer of ROIs, CCL2 and IL-8 in response to LPS [14]. In our study, we demonstrated that CD14^bright^/CD56+ monocytes produce more of the typical monocyte cytokines TNF, IL-10 and IL-23 in response to LPS than CD14^bright^/CD56– monocytes do. This cytokine response to LPS classifies the CD14^bright^/CD56+ monocytes into the inflammatory subset described by Cros *et al*., contrary to the patrolling monocytes which respond to viruses and nucleic acids, but only weakly to LPS [14].

One major finding of the present study is the strong expansion of the CD14^bright^/CD56+ monocyte subset with increasing age in healthy controls. The aging process is associated with age-related changes in the immune system, a phenomenon called *immunosenescence*[15,16]. In RA, characteristic signs of immunosenescence are present in the adaptive immune system, in particular in the T-cell compartment [17]. However, immunosenescence can also be observed in the innate immune system [18]. Monocytic cells of aged mice produce lower amounts of cytokines in response to Toll-like receptor activation [19,20], but older mice have increased cytokine levels in *in vivo* models of sepsis [21,22]. The influence of age on LPS-induced human monocyte cytokine production has been a subject of controversy, with reports ranging from increased to decreased cytokine production [23-26]. In our study, the CD14^bright^/CD56+ monocytes produced more cytokines in response LPS and spontaneously released more ROIs than classical monocytes, but more comprehensive analyses in older people are needed.

Conflicting results have been reported regarding the influence of age on absolute monocyte numbers in the peripheral blood [27,28] and the CD16 monocyte subset composition [23,28,29]. In a previous study of the CD14^bright^/CD16+ monocyte subset in patients with RA, however, we did not observe an age-dependent increase in the CD16+ monocyte subsets in healthy controls and RA patients [6]. In the present study, we observed an increase in the CD14^bright^/CD56+ monocyte subset from 4.7% in healthy controls younger than 30 years of age to 10.2% in healthy controls older than 60 years of age. The CD14^bright^/CD56+ monocyte subset in RA is expanded in younger patients, probably reflecting a preaged innate immune system in RA patients.

In RA, the monocyte subset is dysregulated. In comparison to healthy controls, the absolute number of all monocyte subsets is increased [7,30], and, in relation to the other CD16-defined monocyte subsets, the CD14^bright^/CD16+ monocyte subset is also expanded [6]. Herein we describe another monocyte subpopulation, CD14^bright^/CD56+ monocytes, which is expanded in RA patients. Considering that the CD14^bright^/CD56+ monocyte subset only minimally overlaps the CD14^bright^/CD16+ monocyte subset, RA patients have a major shift toward pathological monocyte subpopulations at the expense of classical monocytes. The expansion of the CD14^bright^/CD56+ monocyte subset in RA patients was not associated with the inflammatory state of the patients, but we did observe a reduction of the subpopulation during anti-TNF treatment. This decrease in the CD14^bright^/CD56+ monocyte frequency was also associated with a better response to the treatment.

## Conclusion

The CD14^bright^/CD56+ monocyte subset is present in healthy controls and expands with increasing age. The frequency of CD14^bright^/CD56+ monocytes is increased in RA patients, declines with effective anti-TNF treatment and is associated with a better response to treatment. CD14^bright^/CD56+ monocytes produce increased cytokine levels in response to LPS and have higher spontaneous ROI production. The CD14^bright^/CD56+ monocyte subset might therefore represent monocytes from a preaged immune system with dysregulated cytokine and ROI production, but further studies are required to confirm this hypothesis.

## Abbreviations

LPS: Lipopolysaccharide; RA: Rheumatoid arthritis; ROI: Reactive oxygen intermediate; TNF: Tumor necrosis factor.

## Competing interests

The authors declare that they have no competing interests.

## Authors’ contributions

MK performed most of the experiments and was involved in data analysis. CB and UW were involved in the study design and data analysis. MR conceived of the project, was involved in the study design and data analysis, and drafted the manuscript. All authors read and approved the final manuscript.
